# Baseline Peripheral Blood Mononuclear Cell Transcriptomics Before Ustekinumab Treatment Is Linked With Crohn's Disease Clinical Response at 1 Year

**DOI:** 10.14309/ctg.0000000000000635

**Published:** 2023-09-01

**Authors:** Maya Granot, Tzipi Braun, Gilat Efroni, Orit Picard, Ella Fudim, Miri Yavzori, Ola Haj, Batia Weiss, Shomron Ben-Horin, Uri Kopylov, Yael Haberman

**Affiliations:** 1 Pediatric Gastroenterology and Nutrition Unit, Sheba Medical Center, Tel-HaShomer, affiliated with the Tel-Aviv University, Tel-Aviv, Israel;; 2 Department of Gastroenterology, Sheba Medical Center, Tel-HaShomer, affiliated with the Tel-Aviv University, Tel-Aviv, Israel;; 3 Cincinnati Children's Hospital Medical Center and the University of Cincinnati College of Medicine, Cincinnati, Ohio, USA.

**Keywords:** Crohn's disease, ustekinumab, transcriptome, gene expression

## Abstract

**INTRODUCTION:**

Ustekinumab, a monoclonal antibody to the p40 subunit of interleukin (IL)-12 and IL-23, is used for Crohn's disease (CD), and the documented clinical remission rate after 1 year was observed in approximately 50% of patients. We aimed to identify predictors for a clinical response using peripheral blood obtained from patients with CD just before ustekinumab treatment initiation.

**METHODS:**

RNA extraction from peripheral blood mononuclear cells was followed by mRNA paired-end sequencing. Differential gene expression was performed using DESeq2.

**RESULTS:**

We processed samples from 36 adults with CD (13 men, 36%) obtained at baseline before starting ustekinumab treatment. Twenty-two of 36 (61%) were defined as responders and 14/36 (39%) as nonresponders after 1 year based on Physician Global Assessment. Differential gene expression between responders (n = 22) and nonresponders (n = 14) did not show a gene expression signature that passed false discovery rate (FDR) correction. However, the analyses identified 68 genes, including CXCL1/2/3, which were induced in nonresponders vs responders with *P* < 0.05 and fold change above 1.5. Functional annotation enrichments of these 68 genes using ToppGene indicated enrichment for cytokine activity (FDR = 1.98E-05), CXCR chemokine receptor binding (FDR = 2.11E-05), IL-10 signaling (FDR = 5.03E-07), genes encoding secreted soluble factors (FDR = 1.73E-05), and myeloid dendritic cells (FDR = 1.80E-08).

**DISCUSSION:**

No substantial differences were found in peripheral blood mononuclear cell transcriptomics between responders and nonresponders. However, among the nonresponders, we noted an increased inflammatory response enriched for pathways linked with cytokine activity and chemokine receptor binding and innate myeloid signature. A larger cohort is required to validate and further explore these findings.

## INTRODUCTION

Crohn's disease (CD) is a chronic relapsing-remitting inflammatory bowel disease (IBD) with a global rise in incidence over the past few decades (1–3). The chronic nature of the disease requires long‐term treatment to induce and maintain remission. Therapeutic options include newer biologics and small molecules. Despite the variety of therapeutic options, response to treatment is still suboptimal due to initial nonresponse, loss of response, or intolerance to currently available treatments (4). It is, therefore, valuable to identify and characterize patients who may respond preferentially to specific therapies. Personalized medicine has the potential to optimize efficacy, decrease the risk of adverse drug events, and reduce costs by establishing the most suitable therapy for a selected patient (5,6). A review summarizing predictors of the primary response to biologic treatment, including anti–tumor necrosis factor (TNF) agents, vedolizumab, and ustekinumab concluded that currently, we do not have any biomarkers that can be used as a predictor of response to biologic treatment in IBD (6). Blood, stool, and tissue were studied for identifying biomarkers, acknowledging the feasibility and simplicity of using blood rather than stool and tissue biomarkers.

Ustekinumab, a monoclonal antibody to the p40 subunit of interleukin (IL)-12 and IL-23, was approved in 2016 for the treatment of moderate-to-severe CD. Clinical remission after 1 year was observed in approximately 50% of patients receiving ustekinumab treatment every 8 weeks (7). Several studies have investigated associations between clinical, biological, or pharmacological parameters and responsiveness to ustekinumab treatment (8–11). We aimed to identify predictors for a clinical response using peripheral blood mononuclear cell (PBMC) transcriptomics obtained from patients with CD just before ustekinumab treatment initiation.

## METHODS

### Cohort

This single-center prospective study included 36 adult patients diagnosed with CD after failing at least 1 line of biologic treatment and before starting ustekinumab treatment. Patients were recruited, and blood sample was drowned before treatment initiation. Written informed consent was obtained from all participants. The design included all consecutive patients with CD starting ustekinumab treatment between May 2017 and May 2021. The recruitment period lasted 4 years, a relatively long time, due to, among others, the COVID-19 pandemic. The decision to start the treatment was made by the treating physician. Inclusion criteria were diagnosis of CD, initiation of treatment with ustekinumab, and failure of previous biological treatments. Demographic, clinical, laboratory, and endoscopic data were retrieved from medical charts. All participants had blood samples processed for PBMC transcriptomics at baseline before ustekinumab treatment initiation. Participants were defined as responders or nonresponders after 1 year based on Physician Global Assessment (PGA). The clinical outcome was a response without steroids (“steroid free”). The treating physician evaluated the patient during a clinic visit and decided whether he or she responded to ustekinumab. The response was considered to have no or mild symptoms regarding abdominal pain, diarrhea, weight loss, bloody stool, fatigue, maintenance of general activity at work or home, general appearance, and abdominal tenderness on physical examination. Nonresponse was considered as having moderate or severe symptoms related to the factors mentioned earlier. The study was approved by the Sheba Medical Center Institutional Review Board.

### RNA extraction and RNA-seq analysis

PBMCs were isolated from the blood sample obtained at baseline just before starting ustekinumab treatment. PBMC samples were isolated from the blood sample freshly collected into ethylenediaminetetraacetic acid tubes. The blood sample was centrifuged at 1,000*g* for 10 minutes at approximately 20 °C, and plasma was extracted. The remaining material was diluted with 2 volumes (approximately 5 mL) of phosphate-buffered saline and transferred to premade Ficoll tube (Leucosep sterile Mat No 163290) and centrifuged at 1,000*g* for 10 minutes. The layer of mononuclear cells was collected and washed with phosphate-buffered saline, and Trizol was added for RNA extraction. RNA extraction from PBMC leukocytes was performed using Qiagen AllPrep RNA/DNA Mini Kit, followed by PolyA-RNA selection, fragmentation, cDNA synthesis, adaptor ligation, TruSeq RNA sample library preparation (Illumina, San Diego, CA), and paired-end 75 bp sequencing. Reads were quantified by kallisto (12) version 42.5 using Gencode v24 as the reference genome. Kallisto output files were summarized to gene level using R package tximport version 1.12.3 (13). Protein-coding genes with transcripts per million (TPM) values above 1 in at least 20% of the samples were used. Differential gene expression between 1-year responders and nonresponders was performed using DESeq2 version 1.34.0 (14). Principal component analysis (PCA) was performed to summarize variations in gene expression between patients. ToppGene/ToppCluster (15,16) platforms were used for functional annotation enrichment analyses and Cytoscape.v3.0.2 (17) for visualization.

### Summary of statistical tests used

Categorical variables were summarized as frequency and percentage and were compared between groups using the χ^2^ test with the Fisher exact test. Continuous variables were analyzed by a 2-tailed Student *t* test or the Mann-Whitney *U* test, as appropriate. A 2-tailed *P* < 0.05 was considered statistically significant. The transcriptomics bioinformatics analyses are described earlier under RNA-seq analysis and these follow previously used methodologies (18–21).

### Study approval

The study was approved by the Sheba Medical Center Institutional Review Board. Informed consent was obtained from all participants.

### Data availability

Data supporting the findings of this study are available from the corresponding author [Y.H.] on reasonable request.

## RESULTS

### Prospective CD cohort

This single-center study included 36 adult patients (13 men, 36%) diagnosed with CD after failure of at least 1 line of biologic treatment before starting ustekinumab treatment. The design included all consecutive patients with CD starting ustekinumab treatment between May 2017 and May 2021. The decision to start the treatment was made by the treating physician. Inclusion criteria were diagnosis of CD, initiation of treatment with ustekinumab, and failure of previous biological treatments. Demographic, clinical, laboratory, and endoscopic data were retrieved from medical charts (Table 1). There were no differences between the groups regarding sex, age, body mass index, smoking, and previous surgeries. The median age was 22 years (interquartile range 19–34) at the diagnosis of CD and 35 years (interquartile range 29–41) at the initiation of Stelara treatment. Twenty-two of 36 (61%) were defined as responders and 14/36 (39%) as nonresponders after 1 year based on PGA. Within the responders, there were more patients with small intestine disease (L1) 82% vs 36%, among nonresponders (*P* = 0.004), with a higher percentage of patients with CD with L3 involvement of the small and large intestine among nonresponders (57% vs 14%, *P* = 0.005). No other significant differences were observed between groups regarding the phenotype, perianal disease, and the number of previous biologic treatments used. At baseline, 35 of the 36 patients had active disease before starting the treatment based on physician assessment, and only 1 patient with CD in the responder's group was switched to ustekinumab due to active psoriasis. We had baseline C-reactive protein measurements for 25 of the 36, of which 18 were above the normal limit, and there was a trend toward a higher fraction of high C-reactive protein in the nonresponders, but this did not reach statistical significance. Baseline calprotectin was available for only 12 of the 36, and its levels did not differ between the responders and nonresponders (Table 1). The clinical outcome was a response without steroids (“steroid free”). The treating physician evaluated the patient during a clinic visit and decided whether he or she responded to ustekinumab. The response was considered to have no or mild symptoms concerning abdominal pain, diarrhea, weight loss, bloody stool, fatigue, maintenance of general activity at work or home, general appearance, and abdominal tenderness on physical examination. Nonresponse was considered as having moderate or severe symptoms related to the abovementioned factors.

**Table 1.** Demographic and clinical characteristics of the study population

	All patients with CD (n = 36)	Responders (n = 22)	Nonresponders (n = 12)	*P* value
Gender, female, n (%)	23 (64)	13 (59)	10 (71)	NS
Age at diagnosis, yr, median (IQR)	22 (19–34)	23 (20–37)	20 (18–23)	NS
Age at inclusion, yr, median (IQR)	35 (29–41)	37 (30–42)	31 (28–39)	NS
BMI, median (IQR)	22 (20–25.5)	24 (20–27)	22 (19.5–22.5)	NS
Smoking, n (%)	5 (14)	3 (14)	2 (14)	NS
Disease location, n (%)				
Small bowel (L1)	23 (64)	18 (82)	5 (36)	**0.004**
Small bowel + colon (L3)	11 (30)	3 (14)	8 (57)	**0.005**
Colon (L2)	2 (6)	1 (4)	1 (7)	NS
Disease duration, yr, median (IQR)	8.1 (4.7–16.1)	6.6 (4.2–15.8)	9.0 (5.7–16.5)	NS
Perianal disease, n (%)	12 (33)	10 (45)	2 (20)	0.053
Disease phenotype, n (%)				NS
Inflammatory (B1)	16 (44)	10 (45)	6 (43)	
Structuring (B2)	7 (19)	6 (27)	1 (7)	
Penetrating (B3)	13 (36)	6 (27)	7 (50)	
Lines of biologics, n (%)^a^				NS
2	17 (47)	13 (59)	4 (29)	
3	12 (33)	6 (27)	6 (43)	
4	6 (17)	3 (8)	3 (21)	
5	1 (3)	0 (0)	1 (7)	
Concomitant therapies, n (%)				NS
None	27 (75.0)	18 (81.8)	9 (64.3)	
Steroids	5 (13.8)	1 (4.5)	4 (28.5)	
Methotrexate	2 (5.5)	1 (4.5)	1 (7.1)	
Azathioprine	1 (2.8)	1 (4.5)	0 (0)	
CDED	1 (2.8)	1 (4.5)	0 (0)	
Prior surgery	16 (44)	10 (45)	6 (43)	NS
N available^b^	N = 25	N = 14	N = 11	0.062
Baseline CRP >5 mg/L (%)	18/26 (73)	8/14 (57)	10/11 (91)	
N available^b^	N = 12	N = 8	N = 4	NS
Baseline fecal calprotectin mic/g, median (IQR)	532 (285–1085)	405 (117–866)	953 (499–1380)	

Bold indicates *P* < 0.05, specific numbers are given for 0.05 < 0 < 0.1.

BMI, body mass index; CD, Crohn's disease; CDED, Crohn's disease exclusion diet; CRP, C-reactive protein; IQR, interquartile range; NS, nonsignificant; TNF, tumor necrosis factor.

a Previous biologics included anti-TNF (infliximab, adalimumab, certolizumab) anti-integrin (vedolizumab).

b Feature was available for the number of patients with CD as indicated.

Ustekinumab nonresponse PBMC, transcriptomics signature, is enriched for pathways linked with cytokine activity and innate myeloid signature.

**Figure 1.**
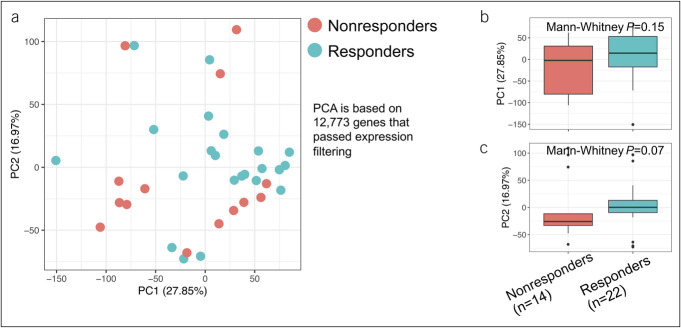
Unsupervised clustering of Crohn's disease peripheral blood mononuclear cell transcriptomics before ustekinumab treatment colored by response after 1 year. (**a**) Principal component analysis (PCA) using the 12,773 genes that passed expression filtering. Values off PC1 (**b**) and PC2 (**c**) stratified by response at 1 year showing a trend in the difference between responders and nonresponders (Mann-Whitney *P* = 0.07).

A total of 12,773 genes passed expression filtering of 1 TPM in 20% in the 36 PBMC samples obtained at baseline just before starting ustekinumab treatment. PCA plot based on these 12,773 genes showed only partial separation between groups (Figure 1).

**Figure 2.**
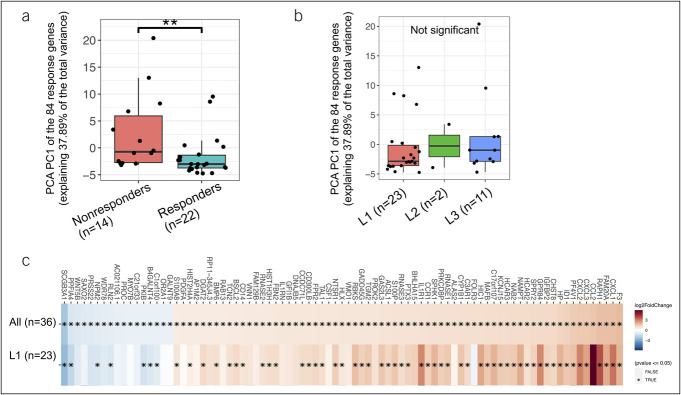
Variation in the expression of the 85 genes linked to ustekinumab response was not linked to disease location. Principal components 1 (PC) that summarized the greatest variation (37.89% of the overall variation) values were extracted. The Crohn’s disease responders significantly different from nonresponders along PC1 values (*P* < 0.007, **a**), while no significant difference was noted when samples were stratified by disease location (L1, L2, L3 in **b**). (**c**) Large fraction of the 84 genes were still significantly differentially expressed when the comparison between responders and nonresponders was performed only within the Crohn's disease group with only ileal involvement (L1).

Differential gene expression between responders (n = 22) and nonresponders (n = 14) did not identify gene expression signature that passed FDR correction of <0.05. However, these analyses identified 85 genes that were differentially expressed with *P* < 0.05 and a fold change difference between the groups that were above 1.5. Sixty-eight genes, including CXCL1/2/3, were induced in nonresponders vs responders, and 17 genes were higher in responders vs nonresponders. The top 10 genes are listed in Table 2, and the complete gene list is summarized in Supplementary Table 1 (see Supplementary Digital Content 1, http://links.lww.com/CTG/B13). PCA was performed to summarize the variation of the 85 genes that differed between responders and nonresponders. Principal component 1 (PC) summarized the greatest variation (37.89% of the overall variation) and the PC1 values per each subject were extracted (Figure 2a,b). The CD responders differ significantly from nonresponders along PC1 values (*P* < 0.007), while no significant difference was noted when samples were stratified by disease location (L1, L2, L3), indicating that the signal that summarized the expression of the 84 genes is not highly linked with disease location. In addition, we specifically looked within the L1 CD group (n = 23, 64% of the overall cohort and the largest group in our cohort). As indicated in Figure 2c, a large fraction of the 84 genes were still significantly differentially expressed when the comparison between responders and nonresponders was performed only within the L1 group. Because L3 had only 11 (with only 3 responders) and L2 had only 2 subjects, we were not able to approach this in these groups.

**Table 2.** Top 10 differentially expressed genes between nonresponders and responders

	Gene	log2FoldChange	Direction of change (nonresponders vs responders)	*P* value
1	F3	1.84	Increased	<0.01
2	CXCL1	1.75	Increased	0.01
3	FAM20A	1.74	Increased	<0.01
4	RAPH1	1.72	Increased	0.01
5	CCL2	1.63	Increased	0.03
6	CXCL3	1.54	Increased	<0.01
7	CXCL2	1.54	Increased	0.01
8	PF4V1	1.49	Increased	<0.01
9	ID1	1.31	Increased	0.02
10	HP	1.25	Increased	0.04

**Figure 3.**
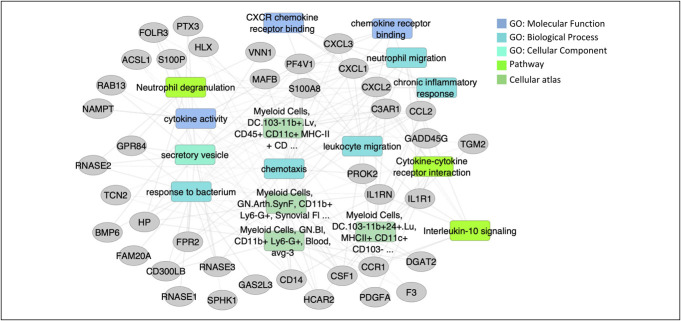
Nonresponders show upregulation of genes enriched for chemokines and myeloid cells. Functional annotation enrichments of the 68 genes induced in peripheral blood mononuclear cell of nonresponders vs responders using ToppGene/ToppCluster and is visualized using Cytoscape.

Functional annotation enrichments of these 68 genes that were induced in nonresponders using ToppGene (Figure 3 and see Supplementary Table 2, Supplementary Digital Content 1, http://links.lww.com/CTG/B13) indicated enrichment for cytokine activity (FDR = 1.98E-05), CXCR chemokine receptor binding (FDR = 2.11E-05), IL-10 signaling (FDR = 5.03E-07), genes encoding secreted soluble factors (FDR = 1.73E-05), myeloid cells, granulocytes CD11b+ (FDR = 6.64E-12), and myeloid cells dendritic cells (FDR = 1.80E-08). By contrast, there were not many functional annotation enrichments for the more limited 17 genes that were upregulated in nonresponders, and those that passed FDR <0.05 are listed in Supplementary Table 2 (see Supplementary Digital Content 1, http://links.lww.com/CTG/B13).

## DISCUSSION

Outcomes for patients starting a new treatment for inflammatory bowel disease are characterized by uncertainty regarding treatment response. With the ongoing incorporation of newer therapeutic options, identifying biomarkers for response are key. However, the identification of markers related to nonresponse is also important because they can highlight pathways that can guide the development of future interventions not approached by the specific therapy. Suboptimal response to medical therapy due to primary nonresponse, secondary loss of response, or intolerance to currently available treatments remains a significant issue (4,22). Biomarkers in peripheral blood are considered easier to obtain compared with fecal and gut mucosal biopsy tissue. In this study, we highlight a subset of 68 genes that are higher in the peripheral blood obtained just before ustekinumab initiation in nonresponders vs responders. Enrichment of these 68 genes that were higher in nonresponders included pathways of chemokines from the CXCL1, 2, 3 family, myeloid cells, granulocytes CD11b+, and dendritic cells.

A significant effort was invested in finding markers to predict treatment response and advance personalized medicine for patients with IBD. Most of the studies focused on discovering biomarkers predicting response to anti-TNF therapy highlighting oncostatin M (23) TNFR2, IL13RA2 (24,25), and TREM1 (26,27) in mucosal biopsies or using blood samples. Our study indicated the induction of the CXCL1, 2, 3 chemokine family in the peripheral blood of nonresponse to ustekinumab treatment. The CXCL1, 2, 3 chemokine family promotes the activation of the innate immune system and the recruitment of neutrophils. Overexpression of these chemokines in intestinal tissue (rather than blood) was already shown to be associated with a lack of response to biological therapy (20,23). More specifically, secondary analyses of the UNIFI trial indicated that IL-22 responsive genes indeed regulated neutrophil recruitment in ulcerative colitis and is associated with resistance to ustekinumab therapy (28), potentially relating to the results highlighted here in nonresponders. These studies overall support an emerging concept in the field that the mucosal inflammatory state, as measured by gene expression, may better define the likelihood of response to current treatment approaches than conventional clinical measures of severity (20).

Our study has several strengths and some limitations. It is a prospective cohort using baseline signal obtained just before initiation of treatment, and it uses real-life 1-year clinical response outcome to ustekinumab treatment. The signal is measured in peripheral blood, which is easier to collect than gut mucosal biopsy tissue. Finally, all participants have failed at least 1 previous biological treatment, representing our “real-life” patients in the clinic, a resistant and hard-to-treat group that will especially benefit from personalized medicine and identification of future targets linked with nonresponse. Limitations include the relatively small sample size and that the response to treatment was defined according to clinical evaluation (PGA) with no biomarker validation. In addition, L3 disease was more common in nonresponders vs responders, which could have been a confounder if L3 disease was associated with nonresponse. However, current evidence indicates lower efficacy of various biological treatments including anti-TNF drugs vedolizumab, ustekinumab, and risankizumab in isolated ileal CD (L1) compared with that in CD of the colon L2/L3 (29). Previous studies regarding specifically the efficacy of ustekinumab treatment in L1 vs L3 locations are scarce and conflicted. Meta-analysis on ustekinumab induction and maintenance trials (7,30) in patients with moderately to severely active CD reported that patients with isolated ileal CD (n = 170) compared with those with colonic CD (n = 136) were significantly less likely to achieve clinical response or remission (31). A retrospective multicenter cohort study in patients with CD achieving steroid-free clinical response to ustekinumab induction therapy (n = 104) demonstrated that colonic disease and ileocolonic disease were associated with lower risk for loss of response during maintenance therapy (32). Another study involving 152 patients with CD showed in a multivariate analysis that only colonic disease predicted clinical response 1 year after ustekinumab initiation (33). By contrast, a study with 407 patients with CD showed that the ileocolonic and colonic diseases were associated with lower clinical response rates at week 26 (34).

A higher fraction of L1 ileal-only CD location in responders might indicate that the differences in gene expression may also relate to disease location; however, as detailed in the result section, no significant difference was noted between responders and nonresponders along PC1 values when samples were stratified by disease location (L1, L2, and L3), indicating that the signal that summarized the expression of the 84 genes is not highly linked with disease location. Nevertheless, it is of interest that a disease that primarily involves the small and large bowel (L3) is linked with higher expression of CXCL1, 2, 3 chemokines in the blood, which promote the activation of the innate immune system and recruitment of neutrophils, and this is linked with less response to ustekinumab treatment. Therefore, disease location may also need to be considered before the selection of a specific biologic therapy. However, a larger cohort is needed to verify and further explore these findings.

## CONFLICTS OF INTEREST

**Guarantor of the article:** Yael Haberman, MD, PhD.

**Specific author contributions:** M.G.: collecting and interpreting data, statistical analysis, writing the article. T.B.: analyzed the data and participated in drafting the article. G.E.: generated the data and participated in drafting the article. O.P. and M.Y.: generated the data and participated in drafting the manuscript. O.H.: recruited patients, gathered data, and participated in drafting the article. B.W. and S.B.-H.: acquisition of data and participated in drafting the article. U.K.: study concept and design, recruited patients, collected data, acquired funding (Jannsen Takeda Medtronic), and participated in drafting the manuscript. Y.H.: study concept and design, analyzed and interpreted the data, and writing of the article. All authors had access to study data and approved the decision to submit the article.

**Financial support:** Research support Jannsen funded this study, but the work was independent, and the company had no influence on the collection, analysis, and interpretation of the data and writing of the article. Other support included the ERC starting grant (Y.H., Grant No. 758313), the Israel Science Foundation (Y.H., Grant No. 785/22), the I-CORE program (Y.H., Grant No. 41/11), and NIDDK P30 DK078392 (Integrative Morphology and Gene Expression Cores).

**Potential competing interests:** U.K.: speaker and advisory fees—Abbvie BMS Celtrion Jannsen Pfizer Roche Takeda Medtronic. All the other authors have no potential competing interests.

**Study Highlights**

**WHAT IS KNOWN**

✓ Therapeutic options for Crohn's disease include new biologic treatments, but response is still suboptimal.
✓ Clinical remission after 1 year of ustekinumab treatment was observed in approximately 50% of patients.
✓ We currently do not have biomarkers to predict response to biological treatment in inflammatory bowel disease.

**WHAT IS NEW HERE**

✓ Clinical nonresponse to ustekinumab treatment is associated with higher baseline expression of CXCL1, 2, 3 chemokine family, which are linked with the recruitment of neutrophils.
✓ Induction of CXCL1, 2, 3 chemokines in intestinal tissue was previously linked with a lack of response to biological therapy, and we show that this signal in the blood at baseline before treatment initiation is also linked with clinical nonresponse to ustekinumab treatment at 1 year.

## Supplementary Material
