# Supplementary material for: Baseline Peripheral Blood Mononuclear Cell Transcriptomics Before Ustekinumab Treatment Is Linked With Crohn's Disease Clinical Response at 1 Year
Source: Clin Transl Gastroenterol. 2023 Sep 1;14(12):e00635. doi: 10.14309/ctg.0000000000000635 (PMC10749706; doi:10.14309/ctg.0000000000000635)
Supplement: Supplementary file 1 [file ct9-14-e00635-s001.docx]

**List of Supplementary Tables**

**Supplementary Table 1:** 85 differentially expressed genes between non-responders vs. responders.

**Supplementary Table 2a:** Table S2a: Functional enrichment of the 68 genes induced in non-responders.

**Supplementary Table 2b**: Functional enrichment of the 17 genes induced in responders.

| Table S1: Differentialy expressed genes between non-responders vs. responders | | | | |
| --- | --- | --- | --- | --- |
|  | Gene | log2FoldChange | Direction  (Higher in non- responders) | P value |
| 1 | F3 | 1.84 | Up | <0.01 |
| 2 | CXCL1 | 1.75 | Up | 0.01 |
| 3 | FAM20A | 1.74 | Up | <0.01 |
| 4 | RAPH1 | 1.72 | Up | 0.01 |
| 5 | CCL2 | 1.63 | Up | 0.03 |
| 6 | CXCL3 | 1.54 | Up | <0.01 |
| 7 | CXCL2 | 1.54 | Up | 0.01 |
| 8 | PF4V1 | 1.49 | Up | <0.01 |
| 9 | ID1 | 1.31 | Up | 0.02 |
| 10 | HP | 1.25 | Up | 0.04 |
| 11 | CHST8 | 1.17 | Up | 0.01 |
| 12 | IGFBP2 | 1.15 | Up | 0.01 |
| 13 | GPR84 | 1.12 | Up | 0.03 |
| 14 | SPRY2 | 1.09 | Up | 0.01 |
| 15 | HCAR2 | 1.07 | Up | 0.00 |
| 16 | NAMPT | 1.07 | Up | 0.04 |
| 17 | NAB2 | 1.06 | Up | 0.02 |
| 18 | HCAR3 | 1.06 | Up | <0.01 |
| 19 | KCNJ15 | 1.06 | Up | 0.01 |
| 20 | C17orf107 | 1.05 | Up | 0.02 |
| 21 | MAFB | 1.04 | Up | 0.02 |
| 22 | HIC1 | 0.99 | Up | 0.02 |
| 23 | FOLR3 | 0.98 | Up | 0.01 |
| 24 | C3AR1 | 0.98 | Up | <0.01 |
| 25 | CYP1B1 | 0.98 | Up | 0.02 |
| 26 | ALAS2 | 0.97 | Up | 0.03 |
| 27 | RNASE1 | 0.96 | Up | 0.02 |
| 28 | PRKCDBP | 0.93 | Up | <0.01 |
| 29 | SPHK1 | 0.91 | Up | 0.02 |
| 30 | CCR1 | 0.91 | Up | 0.03 |
| 31 | IL1R1 | 0.89 | Up | <0.01 |
| 32 | BHLHA15 | 0.86 | Up | 0.03 |
| 33 | PTX3 | 0.85 | Up | 0.01 |
| 34 | RNASE3 | 0.85 | Up | 0.01 |
| 35 | S100P | 0.85 | Up | 0.04 |
| 36 | ACSL1 | 0.83 | Up | 0.03 |
| 37 | GAS2L3 | 0.82 | Up | 0.04 |
| 38 | PROK2 | 0.81 | Up | 0.03 |
| 39 | TGM2 | 0.80 | Up | 0.01 |
| 40 | GADD45G | 0.80 | Up | 0.01 |
| 41 | RBKS | 0.80 | Up | 0.05 |
| 42 | VMO1 | 0.79 | Up | 0.02 |
| 43 | HLX | 0.79 | Up | 0.01 |
| 44 | NTSR1 | 0.77 | Up | 0.03 |
| 45 | CSF1 | 0.77 | Up | 0.01 |
| 46 | TAL1 | 0.76 | Up | 0.02 |
| 47 | FPR2 | 0.76 | Up | 0.01 |
| 48 | CD300LB | 0.75 | Up | 0.02 |
| 49 | CCDC71L | 0.75 | Up | 0.01 |
| 50 | DNAJB5 | 0.75 | Up | 0.04 |
| 51 | GFI1B | 0.75 | Up | 0.03 |
| 52 | IL1RN | 0.74 | Up | 0.05 |
| 53 | FBN2 | 0.72 | Up | 0.02 |
| 54 | HIST1H3H | 0.68 | Up | 0.05 |
| 55 | RNASE2 | 0.67 | Up | 0.01 |
| 56 | FAM129B | 0.67 | Up | 0.03 |
| 57 | VNN1 | 0.67 | Up | 0.04 |
| 58 | CD14 | 0.66 | Up | 0.01 |
| 59 | BSCL2 | 0.64 | Up | 0.01 |
| 60 | TCN2 | 0.64 | Up | 0.02 |
| 61 | RAB13 | 0.63 | Up | 0.01 |
| 62 | BMP6 | 0.62 | Up | 0.04 |
| 63 | RP11-345J4.3 | 0.61 | Up | 0.04 |
| 64 | DGAT2 | 0.60 | Up | 0.03 |
| 65 | AP1M2 | 0.60 | Up | 0.05 |
| 66 | HIST2H4A | 0.60 | Up | <0.01 |
| 67 | PDGFA | 0.59 | Up | 0.03 |
| 68 | S100A8 | 0.59 | Up | 0.03 |
| 69 | GALNT9 | -0.59 | Down | 0.02 |
| 70 | OR2A1 | -0.60 | Down | 0.04 |
| 71 | C1orf100 | -0.60 | Down | 0.01 |
| 72 | B4GALNT4 | -0.61 | Down | 0.03 |
| 73 | PKIB | -0.61 | Down | 0.05 |
| 74 | C21orf33 | -0.62 | Down | 0.01 |
| 75 | MYO7B | -0.62 | Down | 0.05 |
| 76 | PROC | -0.64 | Down | 0.03 |
| 77 | AC021106.1 | -0.66 | Down | 0.02 |
| 78 | RLN2 | -0.70 | Down | 0.01 |
| 79 | WDR78 | -0.75 | Down | 0.01 |
| 80 | NPM2 | -0.78 | Down | 0.02 |
| 81 | PRSS22 | -0.89 | Down | 0.04 |
| 82 | SAXO2 | -0.90 | Down | 0.01 |
| 83 | WNT5B | -0.95 | Down | 0.04 |
| 84 | PPFIA4 | -1.01 | Down | 0.01 |
| 85 | SCGB3A1 | -2.26 | Down | <0.01 |

| Supplementary Table 2a: Functional enrichment of the 68 genes induced in non-responders | | | | |
| --- | --- | --- | --- | --- |
| ID | Name | FDR B&H | Genes from Input | Genes in Annotation |
| GO: Molecular Function | | | |  |
| GO:0005125 | cytokine activity | 0.00001984 | 9 | 250 |
| GO:0030546 | signaling receptor activator activity | 0.00001984 | 12 | 546 |
| GO:0045236 | CXCR chemokine receptor binding | 0.00002109 | 4 | 18 |
| GO:0004522 | ribonuclease A activity | 0.00002109 | 3 | 5 |
| GO:0030545 | signaling receptor regulator activity | 0.00002109 | 12 | 598 |
| GO:0008009 | chemokine activity | 0.00003508 | 5 | 52 |
| GO:0048018 | receptor ligand activity | 0.00004002 | 11 | 536 |
| GO:0005153 | interleukin-8 receptor binding | 0.0001058 | 3 | 9 |
| GO:0016892 | endoribonuclease activity, producing 3'-phosphomonoesters | 0.000171 | 3 | 11 |
| GO:0042379 | chemokine receptor binding | 0.000171 | 5 | 79 |
| GO:0070553 | nicotinic acid receptor activity | 0.0003048 | 2 | 2 |
| GO:0016894 | endonuclease activity, active with either ribo- or deoxyribonucleic acids and producing 3'-phosphomonoesters | 0.001087 | 3 | 21 |
| GO:0005126 | cytokine receptor binding | 0.001961 | 7 | 327 |
| GO:0005102 | signaling receptor binding | 0.002889 | 16 | 1813 |
| GO:0140375 | immune receptor activity | 0.003939 | 5 | 165 |
| GO:0008083 | growth factor activity | 0.005253 | 5 | 178 |
| GO:0004888 | transmembrane signaling receptor activity | 0.006908 | 13 | 1393 |
| GO: Biological Process | | | | |
| GO:0001525 | angiogenesis | 1.536E-09 | 20 | 873 |
| GO:0048514 | blood vessel morphogenesis | 1.536E-09 | 21 | 1029 |
| GO:0006954 | inflammatory response | 1.536E-09 | 21 | 1048 |
| GO:0001944 | vasculature development | 3.216E-09 | 22 | 1239 |
| GO:0001568 | blood vessel development | 5.544E-09 | 21 | 1152 |
| GO:0035239 | tube morphogenesis | 7.619E-09 | 23 | 1467 |
| GO:0002544 | chronic inflammatory response | 8.832E-09 | 7 | 39 |
| GO:0097529 | myeloid leukocyte migration | 5.652E-08 | 12 | 317 |
| GO:0006935 | chemotaxis | 8.094E-08 | 17 | 840 |
| GO:0042330 | taxis | 8.094E-08 | 17 | 842 |
| GO:0009617 | response to bacterium | 8.094E-08 | 20 | 1242 |
| GO:0035295 | tube development | 8.488E-08 | 24 | 1880 |
| GO:0048646 | anatomical structure formation involved in morphogenesis | 8.659E-08 | 24 | 1890 |
| GO:0033993 | response to lipid | 8.659E-08 | 22 | 1567 |
| GO:0040011 | locomotion | 2.582E-07 | 23 | 1834 |
| GO:0030595 | leukocyte chemotaxis | 2.629E-07 | 11 | 302 |
| GO:0060326 | cell chemotaxis | 2.733E-07 | 12 | 389 |
| GO:0032496 | response to lipopolysaccharide | .367E-07 | 14 | 597 |
| GO:0002237 | response to molecule of bacterial origin | 5.613E-07 | 14 | 624 |
| GO:0051240 | positive regulation of multicellular organismal process | 6.336E-07 | 23 | 1950 |
| GO:0097530 | granulocyte migration | 1.845E-06 | 9 | 212 |
| GO:0050900 | leukocyte migration | 2.894E-06 | 12 | 492 |
| GO:0072359 | circulatory system development | 3.904E-06 | 22 | 1977 |
| GO:0002682 | regulation of immune system process | 4.659E-06 | 21 | 1821 |
| GO:0071621 | granulocyte chemotaxis | 5.359E-06 | 8 | 173 |
| GO: Cellular Component | | | | |
| GO:0030141 | secretory granule | 4.752E-06 | 16 | 987 |
| GO:0099503 | secretory vesicle | 8.301E-06 | 17 | 1232 |
| GO:0042581 | specific granule | 0.00002375 | 7 | 160 |
| GO:0034774 | secretory granule lumen | 0.00002375 | 9 | 332 |
| GO:0060205 | cytoplasmic vesicle lumen | 0.00002375 | 9 | 335 |
| GO:0031983 | vesicle lumen | 0.00002375 | 9 | 337 |
| GO:0070820 | tertiary granule | 0.0002804 | 6 | 164 |
| GO:1904724 | tertiary granule lumen | 0.0005605 | 4 | 55 |
| GO:0035580 | specific granule lumen | 0.0008021 | 4 | 62 |
| GO:0031233 | intrinsic component of external side of plasma membrane | 0.008079 | 3 | 48 |
| Interaction |  |  |  |  |
| int:CCL11 | CCL11 interactions | 0.0001371 | 5 | 34 |
| int:XCL2 | XCL2 interactions | 0.0004531 | 4 | 20 |
| int:CXCL3 | CXCL3 interactions | 0.005523 | 3 | 13 |
| int:CCR1 | CCR1 interactions | 0.005523 | 8 | 360 |
| int:CERS6 | CERS6 interactions | 0.005523 | 5 | 97 |

| Pathway |  |  |  |  |  |
| --- | --- | --- | --- | --- | --- |
| ID | Name | Source | FDR B&H | Genes from Input | Genes in Annotation |
| 1457780 | Neutrophil degranulation | BioSystems: REACTOME | 0.00001412 | 13 | 492 |
| M5883 | Genes encoding secreted soluble factors | MSigDB C2 BIOCARTA (v7.5.1) | 0.00001729 | 11 | 343 |
| 1269545 | Class A/1 (Rhodopsin-like receptors) | BioSystems: REACTOME | 0.00007308 | 10 | 322 |
| M42533 | Overview of proinflammatory and profibrotic mediators | MSigDB C2 BIOCARTA (v7.5.1) | 0.00007402 | 7 | 128 |
| 1269546 | Peptide ligand-binding receptors | BioSystems: REACTOME | 0.00007402 | 8 | 188 |
| M9809 | Cytokine-cytokine receptor interaction | MSigDB C2 BIOCARTA (v7.5.1) | 0.00007981 | 9 | 265 |
| 83051 | Cytokine-cytokine receptor interaction | BioSystems: KEGG | 0.00008161 | 9 | 270 |
| M39711 | Cytokines and inflammatory response | MSigDB C2 BIOCARTA (v7.5.1) | 0.0002216 | 4 | 26 |
| 1269576 | G alpha (i) signalling events | BioSystems: REACTOME | 0.0003032 | 8 | 243 |
| M5885 | Ensemble of genes encoding ECM-associated proteins including ECM-affilaited proteins, ECM regulators and secreted factors | MSigDB C2 BIOCARTA (v7.5.1) | 0.000313 | 13 | 751 |
| M5889 | Ensemble of genes encoding extracellular matrix and extracellular matrix-associated proteins | MSigDB C2 BIOCARTA (v7.5.1) | 0.0003689 | 15 | 1026 |
| 1269544 | GPCR ligand binding | BioSystems: REACTOME | 0.0004998 | 10 | 455 |
| 1474301 | IL-17 signaling pathway | BioSystems: KEGG | 0.001491 | 5 | 93 |
| 1269547 | Chemokine receptors bind chemokines | BioSystems: REACTOME | 0.001622 | 4 | 48 |
| 1269566 | Hydroxycarboxylic acid-binding receptors | BioSystems: REACTOME | 0.001858 | 2 | 3 |
| 469200 | Legionellosis | BioSystems: KEGG | 0.002378 | 4 | 55 |
| 812256 | TNF signaling pathway | BioSystems: KEGG | 0.002378 | 5 | 108 |
| 99051 | Chemokine signaling pathway | BioSystems: KEGG | 0.002703 | 6 | 182 |
| M4844 | Chemokine signaling pathway | MSigDB C2 BIOCARTA (v7.5.1) | 0.003163 | 6 | 189 |
| M39477 | Lung fibrosis | MSigDB C2 BIOCARTA (v7.5.1) | 0.003313 | 4 | 63 |
| 1269203 | Innate Immune System | BioSystems: REACTOME | 0.003313 | 15 | 1302 |
| M40067 | SARS-CoV-2 innate immunity evasion and cell-specific immune response | MSigDB C2 BIOCARTA (v7.5.1) | 0.004176 | 4 | 68 |
| M39696 | Peptide GPCRs | MSigDB C2 BIOCARTA (v7.5.1) | 0.005499 | 4 | 75 |
| M22069 | Msp/Ron Receptor Signaling Pathway | MSigDB C2 BIOCARTA (v7.5.1) | 0.005499 | 2 | 6 |
| Coexpression Atlas | | | | | |
| ID | Name | Source | FDR B&H | Genes from Input | Genes in Annotation |
| GSM854338 500 | Myeloid Cells, GN.Arth.SynF, CD11b+ Ly6-G+, Synovial Fluid, avg-3 | Immgen.org, GSE15907 | 6.644E-12 | 17 | 418 |
| GSM854338 100 | Myeloid Cells, GN.Arth.SynF, CD11b+ Ly6-G+, Synovial Fluid, avg-3 | Immgen.org, GSE15907 | 2.124E-11 | 10 | 76 |
| GSM854309 500 | Myeloid Cells, GN.Thio.PC, CD11b+ Ly6-G+, Peritoneal Cavity, avg-3 | Immgen.org, GSE15907 | 1.065E-08 | 14 | 416 |
| GSM854309 100 | Myeloid Cells, GN.Thio.PC, CD11b+ Ly6-G+, Peritoneal Cavity, avg-3 | Immgen.org, GSE15907 | 1.801E-08 | 8 | 74 |
| GSM854258 500 | Myeloid Cells, DC.103-11b+.Salm3.SI, CD45+ MHCII+ Gr1- CD11c-hi CD11b+ CD103- F480+, Small Intestine, avg-4 | Immgen.org, GSE15907 | 1.801E-08 | 13 | 368 |
| bone marrow | bone marrow | Human Protein Atlas | 1.801E-08 | 12 | 292 |
| GSM538239 500 | Myeloid Cells, DC.103-11b+.Lv, CD45+ CD11c+ MHC-II + CD11b high CD103-, Liver, avg-3 | Immgen.org, GSE15907 | 5.717E-08 | 13 | 411 |
| PCBC ctl CardiacMyocyte 1000 | Progenitor-Cell-Biology-Consortium reference CardiacMyocyte top-relative-expression-ranked 1000 | PCBC | 6.679E-08 | 18 | 975 |
| GSM605875 500 | Myeloid Cells, Mo.6C+II-. LN, F480+ Ly6c+ MHCII lo/+, Lymph Node, avg-3 | Immgen.org, GSE15907 | 2.049E-07 | 12 | 374 |
| GSM854262 500 | Myeloid Cells, DC.103-11b+.SI, CD45+ MHCII+ CD11c-hi CD11b+ CD103- F4/80+, Small Intestine, avg-7 | Immgen.org, GSE15907 | 2.275E-07 | 12 | 381 |
| GSM854306 500 | Myeloid Cells, GN.Bl, CD11b+ Ly6-G+, Blood, avg-3 | Immgen.org, GSE15907 | 4.338E-07 | 12 | 409 |
| GSM854312 500 | Myeloid Cells, GN.UrAc.PC, CD11b+ Ly6-G+, Peritoneal Cavity, avg-3 | Immgen.org, GSE15907 | 4.338E-07 | 12 | 410 |
| GSM854303 500 | Myeloid Cells, GN.Arth.BM, CD11b+ Ly6-G+, Bone marrow, avg-3 | Immgen.org, GSE15907 | 5.249E-07 | 12 | 420 |
| GSM854269 500 | Myeloid Cells, DC.103-11b+24+.Lu, MHCII+ CD11c+ CD103- CD11b+ CD24+, Lung, avg-2 | Immgen.org, GSE15907 | 3.876E-06 | 11 | 404 |
| GSM605823 500 | Myeloid Cells, DC.103-11b+.PolyIC.Lu, CD45 MHCII CD11c CD103 CD11b, Lung, avg-3 | Immgen.org, GSE15907 | 4.102E-06 | 11 | 409 |
| GSM854271 500 | Myeloid Cells, DC.103-11b+24-.Lu, MHCII+ CD11c+ CD103- CD11b+ CD24-, Lung, avg-2 | Immgen.org, GSE15907 | 4.199E-06 | 11 | 414 |
| GSM605846 500 | Myeloid Cells, GN.BM, CD11b+ Ly6-G+, Bone marrow, avg-4 | Immgen.org, GSE15907 | 4.199E-06 | 11 | 415 |
| GSM854312 100 | Myeloid Cells, GN.UrAc.PC, CD11b+ Ly6-G+, Peritoneal Cavity, avg-3 | Immgen.org, GSE15907 | 7.586E-06 | 6 | 71 |
| GSM854280 500 | Myeloid Cells, DC.11cloSer.SI, CD45+ MHCII+ CD11c-lo CD11b+, Small Intestine, avg-5 | Immgen.org, GSE15907 | 0.00001669 | 10 | 377 |
| PCBC ctl SmallAirwayEpithel 500 | Progenitor-Cell-Biology-Consortium reference SmallAirwayEpithel top-relative-expression-ranked 500 | PCBC | 0.0000203 | 11 | 493 |
| GSM854322 500 | Myeloid Cells, MF.Medl.SLN, CD11b+ CD169+ F4/80+, Lymph Node, avg-2 | Immgen.org, GSE15907 | 0.00002114 | 10 | 391 |
| GSM538351 500 | B cells, proB.FrA.BM, CD19- IgM- CD43+ CD24- AA4.1+ CD45R+ CD117+ IL7R+, Bone marrow, avg-1 | Immgen.org, GSE15907 | 0.00002217 | 10 | 395 |
| GSM854320 500 | Myeloid Cells, MF.Lv, CD45+ F4/80+ CD11b+, Liver, avg-2 | Immgen.org, GSE15907 | 0.00002492 | 10 | 402 |
| GSM777043 500 | Stromal Cells, Fi. MTS15+.Th, CD45- PDGFRa+ MTS15+, Thymus, avg-3 | Immgen.org, GSE15907 | 0.00006045 | 10 | 445 |
| GSM538280 500 | Myeloid Cells, DC.LC.Sk, MHCII+ CD11b+ CD45+, Skin, avg-2 | Immgen.org, GSE15907 | 0.00006553 | 10 | 451 |
| ToppCell Atlas | | | | | |
| ID | Name | Source | FDR B&H | Genes from Input | Genes in Annotation |
| 22f56ccb81967bff54a1f90e949a8dfc2ce0e4ab | COVID-19 Severe\|World / Disease condition and Cell class | COVID-19 PBMC Myeloid Cell Atlas | 8.432E-31 | 21 | 195 |
| 85714e8da539366b5bce071dfea8f8055de527bc | Transverse-(4) Monocyte\|Transverse / shred on region, Cell type, and subtype | Colon Immune Atlas from Gut Cell Atlas | 8.432E-31 | 21 | 200 |
| 96ecfab57e998134e164b4a62881ef6f2612bd8d | Transverse-Monocyte-Monocyte\|Transverse / Region, Cell class and subclass | Colon Immune Atlas | 8.432E-31 | 21 | 200 |
| d70aeb57b98496474b97906505576640fdbdf34c | Transverse-Monocyte\|Transverse / Region, Cell class and subclass | Colon Immune Atlas | 8.432E-31 | 21 | 200 |
| 0187a4b1d2c3c89b7769d632e6d88190a06b093e | Transverse-(4) Monocyte-(40) Monocyte\|Transverse / shred on region, Cell type, and subtype | Colon Immune Atlas from Gut Cell Atlas | 8.432E-31 | 21 | 200 |
| 00c9c2945d2bdaa128e054cf3c59df86a279b659 | BALF-PFMC-Severe-critical progression d12-22 with-steroid-Myeloid-Monocytic-Classical Monocyte-Mono c3-CD14-VCAN\|Severe-critical progression d12-22 with-steroid / Compartment, severity and other cell annotations on 10x 3' data (130k) | Large Scale COVID-19 Single Cell Data (Ren et al.) | 4.079E-29 | 20 | 193 |
| 9168267a799bbd4111b1227754ab8173ac1a4714 | BALF-PFMC-Severe-critical progression d12-22 with-steroid-Myeloid-Monocytic-Classical Monocyte-Mono c1-CD14-CCL3\|Severe-critical progression d12-22 with-steroid / Compartment, severity and other cell annotations on 10x 3' data (130k) | Large Scale COVID-19 Single Cell Data (Ren et al.) | 4.387E-27 | 19 | 195 |
| 7b61f427ed5ca8ad563e5f112fba950a24c9d956 | BALF-PFMC-Severe-critical progression d12-22 with-steroid-Myeloid-Macrophage-macrophage, alveolar-Macro c2-CCL3L1\|Severe-critical progression d12-22 with-steroid / Compartment, severity and other cell annotations on 10x 3' data (130k) | Large Scale COVID-19 Single Cell Data (Ren et al.) | 4.387E-27 | 19 | 198 |
| 6795ff31f92580195c9db776951f99d647484d24 | mLN-(4) Monocyte\|mLN / shred on region, Cell type, and subtype | Colon Immune Atlas from Gut Cell Atlas | 4.387E-27 | 19 | 199 |
| 241f790cd5d8a9b2451333deecc826a673bae70a | mLN-Monocyte\|mLN / Region, Cell class and subclass | Colon Immune Atlas | 4.387E-27 | 19 | 199 |
| dcee372774c169a3048bdaed3734b3215bf8ffad | mLN-Monocyte-Monocyte\|mLN / Region, Cell class and subclass | Colon Immune Atlas | 4.387E-27 | 19 | 199 |
| bcbb1eee8d79bf670dbe113094c3a292b135ce2f | mLN-(4) Monocyte-(40) Monocyte\|mLN / shred on region, Cell type, and subtype | Colon Immune Atlas from Gut Cell Atlas | 4.387E-27 | 19 | 199 |
| 344801997b89bd81d8d5c3c872934de523233a8d | COVID-19 Severe-Classical Monocyte\|World / disease group, cell group and cell class | PBMC Atlas of COVID-19 Patients (Arunachalam et al.) | 3.521E-25 | 18 | 197 |
| 94e2567f057b2c455a9656b121321a28e7cb1d2b | BALF-PFMC-Severe-critical progression d12-22 with-steroid-Myeloid-Monocytic-Classical Monocyte\|Severe-critical progression d12-22 with-steroid / Compartment, severity and other cell annotations on 10x 3' data (130k) | Large Scale COVID-19 Single Cell Data (Ren et al.) | 3.593E-25 | 18 | 198 |
| a3f22826f3d8f917ff0594bdadd93b60763bcaa2 | severe-CD163+ Monocytes (Sample ID1 d7)\|World / Cohort 1 (10x PBMC) with disease condition, cell group and cell class | PBMC Atlas of COVID-19 Patients (Schulte-Schrepping et al.) | 4.044E-25 | 18 | 200 |
| d78a70f951265ef6df8f64404107be4f53e75d28 | (40) Monocyte\|World / shred on region, Cell type, and subtype | Colon Immune Atlas from Gut Cell Atlas | 6.571E-25 | 18 | 206 |
| 235c24eeab5139f5821fccd5c879d8787682f4ef | 10x 3' v3-lymph node (10x 3' v3)-myeloid-myeloid granulocytic-neutrophil\|lymph node (10x 3' v3) / Per Platform+tissue group, by lineage subgroup, cell group, cell type | Immune and Hematologic Cells from 24 tissues of the Tabula Sapiens: a multiple organ single cell transcriptomic atlas of humans. | 1.271E-23 | 17 | 189 |
| 9f0d956e9a14b1cd2c20a52639c6b22c349b377e | PBMC fresh-frozen-Severe-critical progression d12-22 with-steroid-Myeloid-Monocytic-Classical Monocyte-Mono c1-CD14-CCL3\|Severe-critical progression d12-22 with-steroid / Compartment, severity and other cell annotations on 10x 3' data (130k) | Large Scale COVID-19 Single Cell Data (Ren et al.) | 1.445E-23 | 17 | 191 |
| 2ebb950476767f330241b0077ced22e55e124359 | PBMC fresh-frozen-Severe-critical progression d12-22 with-steroid-Myeloid\|Severe-critical progression d12-22 with-steroid / Compartment, severity and other cell annotations on 10x 3' data (130k) | Large Scale COVID-19 Single Cell Data (Ren et al.) | 1.561E-23 | 17 | 193 |
| a50ee6110ea272d9d9f41d48bb5d43711cc00a32 | 390C-Myeloid-Monocyte-CD14+ Monocyte\|390C / Donor, Lineage, Cell class and subclass (all cells) | Tissue Stability Cell Atlas - Lung Cells | 1.561E-23 | 17 | 193 |
| 580ed65b4152483b642a7e7dbdf00d03588c46f6 | 10x 3' v3-lymph node (10x 3' v3)-myeloid-myeloid monocytic-classical monocyte\|lymph node (10x 3' v3) / Per Platform+tissue group, by lineage subgroup, cell group, cell type | Immune and Hematologic Cells from 24 tissues of the Tabula Sapiens: a multiple organ single cell transcriptomic atlas of humans. | 1.782E-23 | 17 | 195 |
| f3a12a7e23bb4ff6e82fee675dbc090d99587f69 | BALF-PFMC-Severe-critical progression d12-22 with-steroid-Myeloid-Monocytic\|Severe-critical progression d12-22 with-steroid / Compartment, severity and other cell annotations on 10x 3' data (130k) | Large Scale COVID-19 Single Cell Data (Ren et al.) | 2.127E-23 | 17 | 198 |
| 56f9ebf902fe81c590696ba4c88028deaf346841 | (4) Monocyte\|World / shred on Cell type and subtype | Colon Immune Atlas from Gut Cell Atlas | 2.127E-23 | 17 | 198 |
| b078058efc51f0ff4d92123b712933b32824c655 | Caecum-(4) Monocyte-(40) Monocyte\|Caecum / shred on region, Cell type, and subtype | Colon Immune Atlas from Gut Cell Atlas | 2.161E-23 | 17 | 200 |
| 6bfd6ab395e09087aeedc9a7c9ada1b4b8a26194 | Caecum-(4) Monocyte\|Caecum / shred on region, Cell type, and subtype | Colon Immune Atlas from Gut Cell Atlas | 2.161E-23 | 17 | 200 |
| Disease | | | | | |
| ID | Name | Source | FDR B&H | Genes from Input | Genes in Annotation |
| C0033860 | Psoriasis | DisGeNET BeFree | 1.865E-08 | 21 | 1105 |
| C0007222 | Cardiovascular Diseases | DisGeNET BeFree | 4.111E-07 | 22 | 1551 |
| C0004096 | Asthma | DisGeNET BeFree | 4.111E-07 | 23 | 1731 |
| C0003864 | Arthritis | DisGeNET BeFree | 4.111E-07 | 18 | 992 |
| C0009324 | Ulcerative Colitis | DisGeNET BeFree | 4.111E-07 | 20 | 1277 |
| C0021390 | Inflammatory Bowel Diseases | DisGeNET BeFree | 4.111E-07 | 21 | 1437 |
| C0003850 | Arteriosclerosis | DisGeNET BeFree | 6.464E-07 | 24 | 1995 |
| C0004153 | Atherosclerosis | DisGeNET BeFree | 6.464E-07 | 24 | 1998 |
| C0032285 | Pneumonia | DisGeNET BeFree | 3.543E-06 | 16 | 908 |
| C1800706 | Idiopathic Pulmonary Fibrosis | DisGeNET BeFree | 3.543E-06 | 15 | 786 |
| C3714636 | Pneumonitis | DisGeNET BeFree | 0.00000436 | 14 | 682 |
| C1519670 | Tumor Angiogenesis | DisGeNET BeFree | 5.362E-06 | 15 | 822 |
| C0010054 | Coronary Arteriosclerosis | DisGeNET BeFree | 6.418E-06 | 18 | 1262 |
| C0010068 | Coronary heart disease | DisGeNET BeFree | 6.982E-06 | 19 | 1432 |
| C0024299 | Lymphoma | DisGeNET BeFree | 8.887E-06 | 19 | 1460 |
| C1719672 | Severe Sepsis | DisGeNET BeFree | 9.308E-06 | 8 | 168 |
| C0025500 | Mesothelioma | DisGeNET BeFree | 9.904E-06 | 12 | 520 |
| C0031099 | Periodontitis | DisGeNET BeFree | 0.00001052 | 13 | 639 |
| C1956346 | Coronary Artery Disease | DisGeNET BeFree | 0.00001126 | 19 | 1504 |
| C0009319 | Colitis | DisGeNET BeFree | 0.00002141 | 16 | 1094 |
| C0023890 | Liver Cirrhosis | DisGeNET BeFree | 0.00002161 | 16 | 1099 |
| C0027051 | Myocardial Infarction | DisGeNET BeFree | 0.00002161 | 19 | 1582 |
| C0036690 | Septicemia | DisGeNET BeFree | 0.00002643 | 17 | 1279 |
| C0001430 | Adenoma | DisGeNET BeFree | 0.00002643 | 16 | 1126 |
| C0026764 | Multiple Myeloma | DisGeNET BeFree | 0.00004589 | 19 | 1674 |

|  | | | | |
| --- | --- | --- | --- | --- |
| Supplementary Table 2b: Functional enrichment of the 17 genes induced in responders | | | | |
| ID | Name | FDR B&H | Genes from Input | Genes in Annotation |
| GO: Molecular Function | | | |  |
| GO:0008376 | acetylgalactosaminyltransferase activity | 0.02163 | 2 | 35 |
| GO:0033842 | N-acetyl-beta-glucosaminyl-glycoprotein 4-beta-N-acetylgalactosaminyltransferase activity | 0.049 | 1 | 2 |
| GO: Biological Process: No results to display | | | | |
| GO: Cellular Component: No results to display | | | | |
| Interaction: No results to display | | | | |
| Co Expression Atlas; No results to display | | | | |
| ToppCell Atlas | | | | |
| ba64bfb757d6e3be631ee95abfa24b47861f6a14 | BALF-PFMC-Severe-critical progression d12-22 with-steroid-Myeloid-Dendritic-conventional dendritic cell\|Severe-critical progression d12-22 with-steroid / Compartment, severity and other cell annotations on 10x 3' data (130k) | 0.003055 | 4 | 179 |
| e9ff4398e880d13894e52c0ba9cf19a7662c248b | 3'-Distal airway\|3' / 5'-vs-3', Tissue groups, Lineages, Lineage subclass, Cell type2, Cell subtype2 L4.5 | 0.003055 | 4 | 194 |
| 4e25e6362ef793b08de2f4d1dccb6b1fc9c072a5 | Club cells-Donor 05\|World / lung cells shred on cell class, cell subclass, sample id | 0.009098 | 3 | 164 |
| cfe07a1ebed8aecb08d0476db71fe314998d54aa | droplet-Lung-3m-Hematologic-myeloid-plasmacytoid dendritic cell-plasmacytoid dendritic\|3m / method, tissue, age, lineage, sublineage, cell ontologies, cell type and subtype | 0.009098 | 3 | 164 |
| 6d331e74efecb4733d87653f86cd67247be05025 | droplet-Lung-3m-Hematologic-myeloid-plasmacytoid dendritic cell\|3m / method, tissue, age, lineage, sublineage, cell ontologies, cell type and subtype | 0.009098 | 3 | 164 |
| 8e5a262dc4f04c5fc75b9a7a907832a0d7877df0 | droplet-Bladder-BLADDER-1m-Hematologic-granulocyte-monocyte progenitor\|Bladder / Skin Bladder Kidney Mammary Gland - method, tissue, subtissue, age, lineage, cell ontology and free annotation | 0.009098 | 3 | 166 |
| 22c89d4eb44573f6301069ec7ca7cbd0dd958403 | droplet-Liver-Npc-18m-Myeloid-macrophage/monocyte\|Liver / Large Intestine Pancreas Liver - method, tissue, subtissue, age, lineage, cell ontology and free annotation | 0.009098 | 3 | 168 |
| 4e1b522c7400c9d87f63c00e4e1dd07136095cd6 | 3'-GW trimst-2-LargeIntestine-Epithelial-neuro-epithelial-EC cells (NPW+)\|GW trimst-2 / Celltypes from developing, pediatric, Crohn's, & adult GI tract | 0.009098 | 3 | 168 |
| ce5e92a1df3acfa5cd8d5a134e4af231dc592a13 | droplet-Liver-Npc-18m-Myeloid-myeloid leukocyte\|Liver / Large Intestine Pancreas Liver - method, tissue, subtissue, age, lineage, cell ontology and free annotation | 0.009098 | 3 | 168 |
| 18cd941bf3ca7cc0b6af1317bd2619d283353de0 | facs-Marrow-B-cells-18m-Myeloid-monocyte + promonocyte\|Marrow / Spleen Marrow Thymus - method, tissue, subtissue, age, lineage, cell ontology and free annotation | 0.009098 | 3 | 170 |
| 6e82e41acd1299aa549a32ac036b39754abdf376 | facs-Marrow-B-cells-18m-Myeloid-promonocyte\|Marrow / Spleen Marrow Thymus - method, tissue, subtissue, age, lineage, cell ontology and free annotation | 0.009098 | 3 | 170 |
| 0b9912f07393aca889db72b40da08e0bc9bc1e8c | AT1-AT2 cells-IPF 03\|World / lung cells shred on cell class, cell subclass, sample id | 0.009098 | 3 | 171 |
| 207aa0118633cbe9a65839bbb1bb3ba9f8118ad2 | 3'-Broncho-tracheal-Epithelial-Epithelial transtional-secretory-nasal mucosa goblet cell-Goblet (nasal)-Goblet (nasal) L.0.2.2.2\|3' / 5'-vs-3', Tissue groups, Lineages, Lineage subclass, Cell type2, Cell subtype2 L4.5 | 0.009098 | 3 | 174 |
| 38dec78efc99fe23479cba68cfa441e62bd6a08b | E16.5-Epithelial-Epithelial Airway-Neurosecretory\|E16.5 / Age group by Lineage, Lineage subclass, Cell type, subtypes-by-prolif | 0.009098 | 3 | 175 |
| 510c46bfd250ef83fcad8f6b01efbcca9d71a9a2 | droplet-Marrow-nan-18m-Myeloid-monocyte\|Marrow / Spleen Marrow Thymus - method, tissue, subtissue, age, lineage, cell ontology and free annotation | 0.009098 | 3 | 175 |
| dee780cfa85234a7cd7bf440b66b84cec959893e | 3'-Broncho-tracheal-Epithelial-Epithelial transtional-secretory-nasal mucosa goblet cell-Goblet (nasal)-Goblet (nasal) L.0.2.2.1\|3' / 5'-vs-3', Tissue groups, Lineages, Lineage subclass, Cell type2, Cell subtype2 L4.5 | 0.009098 | 3 | 176 |
| ed788a5969edfd1199828ca5b0dd34e7f29c4d30 | facs-Trachea-nan-18m-Epithelial-ciliated columnar cell of tracheobronchial tree\|Trachea / Lung Trachea - method, tissue, subtissue, age, lineage, cell ontology and free annotation | 0.009098 | 3 | 176 |
| 87de432c680b80555342afac864599bbc002c040 | 5'-Airway Nasal-Epithelial-Airway ciliated-ciliated columnar cell of tracheobronchial tree-Multiciliated (non-nasal)-\|5' / 5'-vs-3', Tissue groups, Lineages, Lineage subclass, Cell type2, Cell subtype2 L4.5 | 0.009098 | 3 | 176 |
| 3a94ee9b58ebdbbc75e00954cf1f6922f88f8f88 | BALF-PFMC-Severe-critical progression d12-22 with-steroid-Myeloid-Dendritic-conventional dendritic cell-DC c2-CD1C\|Severe-critical progression d12-22 with-steroid / Compartment, severity and other cell annotations on 10x 3' data (130k) | 0.009098 | 3 | 177 |
| 89f0537459a321ded956bf70ad56fefc0854f8b0 | facs-Lung-3m-Hematologic-myeloid-myeloid dendritic cell-dendritic cell\|3m / method, tissue, age, lineage, sublineage, cell ontologies, cell type and subtype | 0.009098 | 3 | 178 |
| e520eadde4f1b7fb868dbbe94c4d62b3776c55eb | facs-Lung-Endomucin-24m-Myeloid-myeloid dendritic cell\|Lung / Lung Trachea - method, tissue, subtissue, age, lineage, cell ontology and free annotation | 0.009098 | 3 | 179 |
| d4efbc34f52136039b96451fd0b0a0ad164197c6 | facs-Trachea-18m-Epithelial-airway epithelial-tracheobronchial ciliated cell-ciliated cell\|18m / method, tissue, age, lineage, sublineage, cell ontologies, cell type and subtype | 0.009098 | 3 | 179 |
| 8a66d197a2f55d763ff7ef0bec89ee96f59c3937 | facs-Trachea-18m-Epithelial-airway epithelial-tracheobronchial ciliated cell\|18m / method, tissue, age, lineage, sublineage, cell ontologies, cell type and subtype | 0.009098 | 3 | 179 |
| 263937906ddabc798bbe60f0da28ba859a5c72e2 | 3'-Pediatric IBD-SmallIntestine-Epithelial-Tuft-related\|Pediatric IBD / Celltypes from developing, pediatric, Crohn's, & adult GI tract | 0.009098 | 3 | 183 |
| bcb3ac9f9b51346e0a2b0bda96d149b3c11f7883 | droplet-Marrow-BM-30m-Myeloid-promonocyte\|Marrow / Spleen Marrow Thymus - method, tissue, subtissue, age, lineage, cell ontology and free annotation | 0.009098 | 3 | 186 |
| Disease: | | |  |  |
| cv:C2674321 | Thrombophilia due to protein C deficiency, autosomal dominant | 0.0005372 | 1 | 1 |
| cv:C2676759 | Thrombophilia due to protein C deficiency, autosomal recessive | 0.0005372 | 1 | 1 |
| 176860 | THROMBOPHILIA DUE TO PROTEIN C DEFICIENCY, AUTOSOMAL DOMINANT | 0.0005372 | 1 | 1 |
| 612304 | THROMBOPHILIA DUE TO PROTEIN C DEFICIENCY, AUTOSOMAL RECESSIVE | 0.0005372 | 1 | 1 |
| cv:C3160733 | Thrombophilia due to thrombin defect | 0.003436 | 1 | 8 |
| cv:CN120488 | Anophthalmia-microphthalmia syndrome | 0.007507 | 1 | 21 |
